# Radiomics and Artificial Intelligence Analysis with Textural Metrics Extracted by Contrast-Enhanced Mammography in the Breast Lesions Classification

**DOI:** 10.3390/diagnostics11050815

**Published:** 2021-04-30

**Authors:** Roberta Fusco, Adele Piccirillo, Mario Sansone, Vincenza Granata, Maria Rosaria Rubulotta, Teresa Petrosino, Maria Luisa Barretta, Paolo Vallone, Raimondo Di Giacomo, Emanuela Esposito, Maurizio Di Bonito, Antonella Petrillo

**Affiliations:** 1 Radiology Division, Istituto Nazionale Tumori-IRCCS-Fondazione G. Pascale, 80131 Naples, Italy; r.fusco@istitutotumori.na.it (R.F.); m.rubulotta@istitutotumori.na.it (M.R.R.); t.petrosino@istitutotumori.na.it (T.P.); m.barretta@istitutotumori.na.it (M.L.B.); p.vallone@istitutotumori.na.it (P.V.); a.petrillo@istitutotumori.na.it (A.P.); 2 Department of Electrical Engineering and Information Technologies, Università degli Studi di Napoli Federico II, 80125 Naples, Italy; adelep.92@hotmail.it (A.P.); msansone@unina.it (M.S.); 3 Senology Surgical Division, Istituto Nazionale Tumori–IRCCS-Fondazione G. Pascale, 80131 Naples, Italy; r.digiacomo@istitutotumori.na.it (R.D.G.); emanuela.esposito@istitutotumori.na.it (E.E.); 4 Pathology Division, Istituto Nazionale Tumori–IRCCS-Fondazione G. Pascale, 80131 Naples, Italy; m.dibonito@istitutotumori.na.it

**Keywords:** breast, contrast-enhanced digital mammography, radiomics, artificial intelligence

## Abstract

The aim of the study was to estimate the diagnostic accuracy of textural features extracted by dual-energy contrast-enhanced mammography (CEM) images, by carrying out univariate and multivariate statistical analyses including artificial intelligence approaches. In total, 80 patients with known breast lesion were enrolled in this prospective study according to regulations issued by the local Institutional Review Board. All patients underwent dual-energy CEM examination in both craniocaudally (CC) and double acquisition of mediolateral oblique (MLO) projections (early and late). The reference standard was pathology from a surgical specimen for malignant lesions and pathology from a surgical specimen or fine needle aspiration cytology, core or Tru-Cut needle biopsy, and vacuum assisted breast biopsy for benign lesions. In total, 104 samples of 80 patients were analyzed. Furthermore, 48 textural parameters were extracted by manually segmenting regions of interest. Univariate and multivariate approaches were performed: non-parametric Wilcoxon–Mann–Whitney test; receiver operating characteristic (ROC), linear classifier (LDA), decision tree (DT), k-nearest neighbors (KNN), artificial neural network (NNET), and support vector machine (SVM) were utilized. A balancing approach and feature selection methods were used. The univariate analysis showed low accuracy and area under the curve (AUC) for all considered features. Instead, in the multivariate textural analysis, the best performance considering the CC view (accuracy (ACC) = 0.75; AUC = 0.82) was reached with a DT trained with leave-one-out cross-variation (LOOCV) and balanced data (with adaptive synthetic (ADASYN) function) and a subset of three robust textural features (MAD, VARIANCE, and LRLGE). The best performance (ACC = 0.77; AUC = 0.83) considering the early-MLO view was reached with a NNET trained with LOOCV and balanced data (with ADASYN function) and a subset of ten robust features (MEAN, MAD, RANGE, IQR, VARIANCE, CORRELATION, RLV, COARSNESS, BUSYNESS, and STRENGTH). The best performance (ACC = 0.73; AUC = 0.82) considering the late-MLO view was reached with a NNET trained with LOOCV and balanced data (with ADASYN function) and a subset of eleven robust features (MODE, MEDIAN, RANGE, RLN, LRLGE, RLV, LZLGE, GLV_GLSZM, ZSV, COARSNESS, and BUSYNESS). Multivariate analyses using pattern recognition approaches, considering 144 textural features extracted from all three mammographic projections (CC, early MLO, and late MLO), optimized by adaptive synthetic sampling and feature selection operations obtained the best results (ACC = 0.87; AUC = 0.90) and showed the best performance in the discrimination of benign and malignant lesions.

## 1. Introduction

Breast cancer is the most common female disease in the word, diagnosed primarily in over-fifties women [1,2,3].

Mammography (MX), introduced in the 1960s, plays a pivotal role in cancer screening, detection, and follow-up, despite the availability of various other breast imaging modalities, such as ultrasound (US) and breast Magnetic Resonance Imaging (MRI), due to its properties and qualities [4,5].

Contrast-enhanced mammography (CEM) is a diagnostic technique that combines the advantages of standard full-field digital mammography (FFDM) with the intravenous administration of an iodinated contrast medium, highlighting neovascularity associated with actively growing malignancy. The use of contrast agents in cancer detection is based on the phenomenon that neoplasms induce angiogenesis for further tumor growth. Contrast medium can pass through the walls of new rapidly formed vessels into the (tumor) interstitium, causing enhancement.

In addition, dual-energy CEM is a quick and well-tolerated examination that eliminates breast density as a limiting factor when interpreting two-dimensional (2D) mammograms by utilizing a dual-energy acquisition system, which then generates a subtracted image to outline areas of enhancement.

Previous studies have evaluated the sensitivity of CEM compared to conventional digital MX, US, and MRI [6,7,8]. CEM sensitivity has been reported in the range 90–100% [9], much higher in comparison to MX and US alone [8]. CEM allows to identify additional occult cancers at mammography, to assess more accurately the disease extent, and to guide surgical and treatment planning [8,9,10,11].

However, several studies have reported that CEM sensitivity increases compared to conventional MX at the expense of specificity that ranges in the interval of 58–70% [12]. This issue could be resolved using quantitative feature extracted by CEM images.

Recent significant advancements within the field of medical image analysis relies on the application of Artificial Intelligence (AI) methods for the processing of large quantities of data from different imaging modalities [13,14].

Radiomics is the process of extracting quantitative properties, named features, from images. This feature extraction activity is typically realized by means of pattern recognition algorithms and provides, as a result, a set of numbers, each one representing a quantitative description of a specific either geometrical or physical property of the image portion under consideration. In oncological applications, examples of features are tumor size, shape, intensity, and texture, collectively providing a comprehensive tumor characterization, called the radiomics signature of the tumor [15,16,17].

The texture features analysis characterize intensity histogram and the relationships between different intensity levels within lesion; moreover, it describe spatial and spectral frequency patterns [18,19,20].

Several authors proposed radiomics analysis for breast cancer detection and classification, based on lesion heterogeneity information (textural features) and opportune classifiers [21,22,23,24,25,26,27,28,29,30,31,32].

This work has the objective to estimate the diagnostic accuracy of textural features extracted by dual-energy CEM images by carrying out univariate and multivariate statistical analyses, using artificial intelligence approaches in the classification of benign and malignant breast lesions.

## 2. Methods

### 2.1. Patient Selection

From October 2017 to April 2018, 80 consecutive patients with known unifocal and or multifocal/multicentric breast lesions (mean age ± standard deviation of 50.7 ± 11.6 years (range 26–78)) were enrolled in this prospective study according to regulations issued by the local Institutional Review Board. All women gave their written informed consent for research purposes. Inclusion criteria: patient with known breast lesions, histologically proven, and patient underwent dual-energy CEM examination in both craniocaudal (CC) and mediolateral oblique (MLO) projections.

Exclusion criteria were breast implants, pregnancy or possible pregnancy, a contraindication to the intravenous use of iodinated or gadolinium-chelated contrast agent (e.g., allergy to either agent or severely impaired renal function), inability to keep upright immobility during the examination, neoadjuvant chemotherapy treatment, hormone treatment (the most common forms of hormone therapy for breast cancer work by blocking estrogen hormones from attaching to receptors on cancer cells or by decreasing the body’s production of hormones) or radiation therapy at the time of imaging.

Overall, 104 suspected breast lesions of 80 enrolled patients were analyzed. A total of 19 patients were excluded: 6 for contraindication to the intravenous use of iodinated or gadolinium-chelated contrast agent; 4 for neoadjuvant chemotherapy treatment; 4 for breast implants; 2 for inability to keep upright immobility during the examination; and 3 for hormone treatment or radiation therapy.

### 2.2. Imaging Protocol

All CEM examinations were performed with a dual-energy mammography system (Hologic’s Selenia^®^ Dimensions^®^ Unit, Bedford, MA, USA). Approximately two minutes after an intravenous injection of 1.5 mL/kg body weight of iodinated contrast medium (Visipaque 320; GE Healthcare, Inc, Princeton, NJ, USA) at a rate of 2–3 mL/s, each woman was properly positioned in a CC view.

Two images were obtained per each breast through a low-energy exposure (26–31 kVp, according to breast thickness and density) and a high-energy exposure (45–49 kVp), straddling the k-edge of iodine. The low-energy image served as the equivalent of a two-dimensional FFDM examination, which was previously demonstrated [33,34,35]. The high-energy image is not diagnostic and is used for post-processing purposes. The total X-ray dose delivered to the patient for a pair of low- and high-energy images was estimated to be between 0.7 and 3.6 mGy, depending on breast thickness (30 to 80 mm) and tissue composition (0 to 100% glandular tissue); it is approximately 1.2-times the dose delivered in a standard single-view digital mammography [33,34,35].

**Figure 1:**
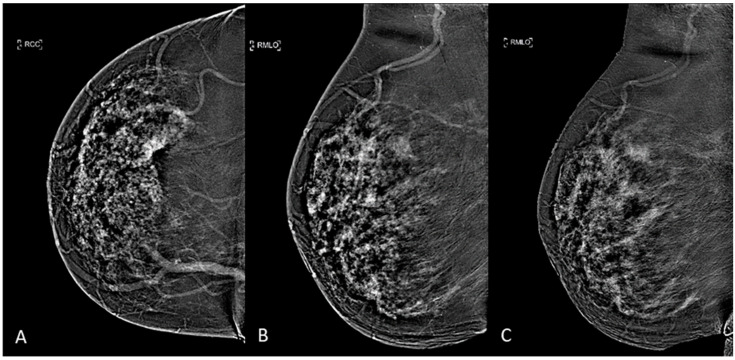
CEM subtracted images of a multifocal IDC in a 53-years-old woman with dense breast: (**A**) CC view, (**B**) early-MLO view, (**C**) late-MLO view.

The two previously acquired images are then digitally subtracted of each other to produce a resultant recombined image that highlights contrast enhancement uptake area and gives functional information [10], as it can be seen in Figure 1. Four and eight minutes after contrast agent administration, each breast were compressed in the MLO view: early-MLO and late-MLO view images were respectively acquired (Figure 1).

### 2.3. Histopathological Analysis

The reference standard was histopathologic examination. Overall, 104 samples of 80 patients were analyzed histopathologically: 65 surgical specimens for malignant lesions and 39 samples (surgical specimen or fine needle aspiration cytology (FNAC), core or Tru-Cut needle biopsy, and vacuum assisted breast biopsy (VABB)) for benign lesions. Histopathological examination was performed before CEM examination. This procedure did not change the shape of the lesion and therefore of the segmentation of the lesions.

Tumor stages were classified according to the system implemented by the American Joint Committee on Cancer staging. Ductal carcinoma in situ and invasive cancer tumors were counted as malignant lesions including, also, the papillary breast cancer that is a very rare type of invasive ductal breast cancer that accounts for fewer than 1% of all breast cancers.

All other results, including fibroadenoma, ductal hyperplasia, dysplasia, cysts, fibrosis and adenosis, were considered non-malignant lesions (Table 1).

### 2.4. Image Processing

Two expert radiologists, with 22 and 15 years of experience respectively, performed segmentation by drawing manually the regions of interest (ROIs) first separately and then together and in accordance with each other.

**Figure 2:**
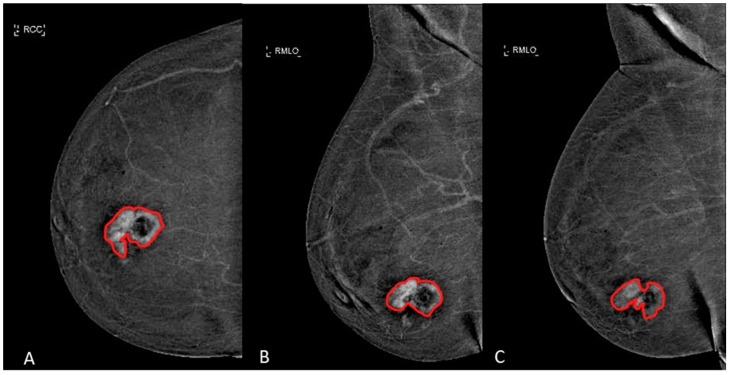
Manual segmentation (red line) of an ILC in a 58-years-old woman with dense breast in (**A**) CC view, (**B**) early MLO view and (**C**) late MLO view.

The margins of the breast lesions were defined on dual-energy subtracted (DES) images, where contrast uptake was emphasized, both in CC and in early and late MLO view. An example is shown in Figure 2.

DES images of each woman were processed in MATLAB (The MathWorks, Inc., Natik, MA, USA [36]) and for each ROI radiomics textural features were extracted. They provide information in the spatial distribution of intensity levels in a neighborhood.

The Texture Toolbox of MATLAB^®^ realized by Vallières et al. [37], which includes 48 parameters, calculated according to the Image Biomarker Standardization Initiative [38], was considered. The package implements wavelet band-pass filtering, isotropic resampling, discretization length corrections and different quantization tools. The toolbox can be downloaded at https://it.mathworks.com/matlabcentral/fileexchange/51948-radiomics (accessed on 15 April 2019).

The textural features (Table 2) include both first-order features and second-order ones; an extra detailed description for each one has been provided in the Appendix A.

### 2.5. Statistical Analysis

The statistical analysis included univariate and multivariate approaches and were performed with the RStudio software [39].

#### 2.5.1. Univariate Analysis

The intra-class correlation coefficient (ICC) was calculated to assess the robustness of manual segmentation for the radiomics features obtained considering the two volumes of interest segmented separately by two expert radiologists.

For two-groups comparisons, we used the non-parametric Wilcoxon–Mann–Whitney test for continuous variables. Receiver operating characteristic (ROC) analysis was performed.

To individuate the optimal cut-off value for each feature the Youden index was calculated. Area under ROC curve (AUC), sensitivity (SENS), specificity (SPEC), positive predictive value (PPV), negative predictive value (NPV), and accuracy (ACC) were obtained considering the optimal cut-off values identified maximizing the Youden index [40].

A *p*-value < 0.05 was considered as significant. However, false discovery rate (FDR) adjustment according to Benjamini and Hochberg for multiple testing was considered.

#### 2.5.2. Multivariate Analysis

Multivariate analysis was carried out following the pattern recognition model and using linear classifier (linear discriminant analysis—LDA), decision tree (TREE), *k*-nearest neighbours (KNN), artificial neural network (NNET), and support vector machine (SVM) to assess the diagnostic accuracy using all extracted metrics of textural parameters. In recent years, several authors have deepened the subject in their work [18,41,42,43,44]. Moreover, a brief description of each classifier has already been discussed in a previous article [14].

Configuration settings for each classifier are provided in Table 3.

The classification analysis has been cross-validated using 10-fold cross validation (10-fold CV) and the Leave-one-out cross validation (LOOCV) approaches and median values of AUC, accuracy, sensitivity, specificity, PPV e NPV were obtained.

Each classifier received the same set or subset of robust features, identified by a feature selection with the least absolute shrinkage and selection operator (LASSO) method [45]. In the LASSO method, 10-fold cross-validation was used to select the optimal regularization parameter alpha, as the average of mean square error of each patient was the smallest. With the optimal alpha, features with a non-zero coefficient in LASSO were reserved. Note that the shrinkage requires the selection of a tuning parameter (lambda) that determines the amount of penalization. Feature selection was carried out considering both the λ value with the minimum Mean Squared Error (minMSE) and the largest λ value within one standard error of it (1SE) [46].

In addition, the presence of less-represented classes has led the use of two techniques to synthesize data and, therefore, to help balance the classes (malignant and benign) giving the possibility to better train the considered classifiers: the self-adaptive synthetic over-sampling (SASYNO) approach and the adaptive synthetic sampling (ADASYN) approach. This allowed to boost the performance both overall and in terms of confusion matrix [47,48,49,50,51].

A brief informal description of LASSO method parameters and of SASYNO and ADASYN functions has already been discussed previously [52].

The best model was chosen considering the highest area under the ROC curve and highest accuracy.

## 3. Results

The ICC for radiomics textural features was excellent (median value 0.92, range 0.87–0.98), for all extracted features by DCE-MRI.

Table 4 reports the diagnostic accuracy of textural parameters for CC and early and late MLO view in terms of AUC and *p*-value.

**Figure 3:**
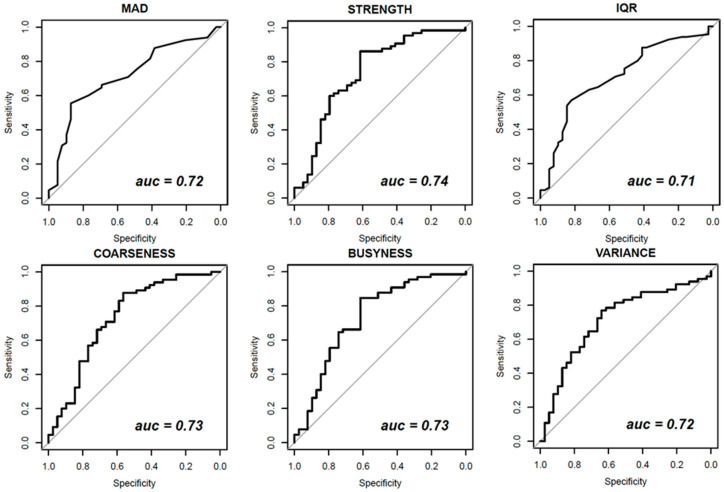
Receiver Operating Characteristic (ROC) curves of significant textural features with AUC values for CC view.

**Figure 4:**
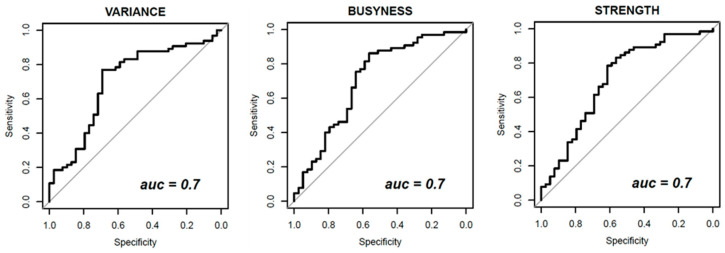
Receiver Operating Characteristic (ROC) curves of significant textural features with AUC values for early-MLO view.

**Figure 5:**
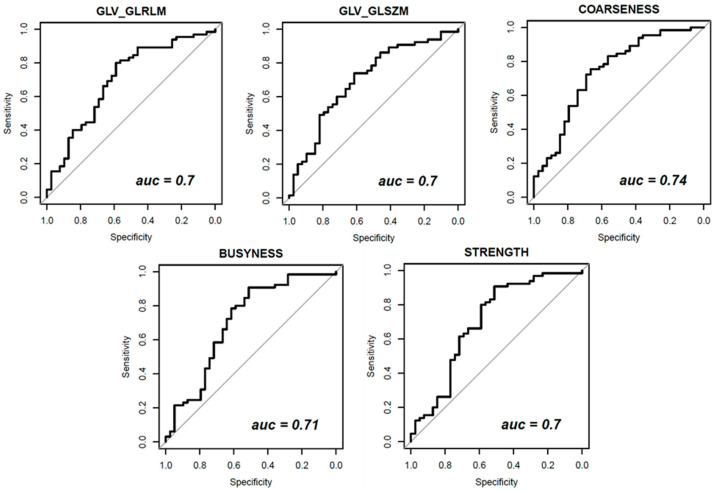
Receiver Operating Characteristic (ROC) curves of significant textural features with AUC values for late-MLO view.

Figure 3, Figure 4 and Figure 5 show ROC curves trend of significant textural features: MAD, IQR, VARIANCE, COARSNESS, BUSYNESS, and STRENGHT for CC projection, VARIANCE, BUSYNESS and STRENGHT for early-MLO projection and GLV_RLRLM, GLV_GLSZM, COARSNESS, BUSYNESS, and STRENGHT for late-MLO projection.

Considering these results, the univariate analysis showed low accuracy and area under the curve for all considered features in the differentiation of benign and malignant lesions.

**Figure 6:**
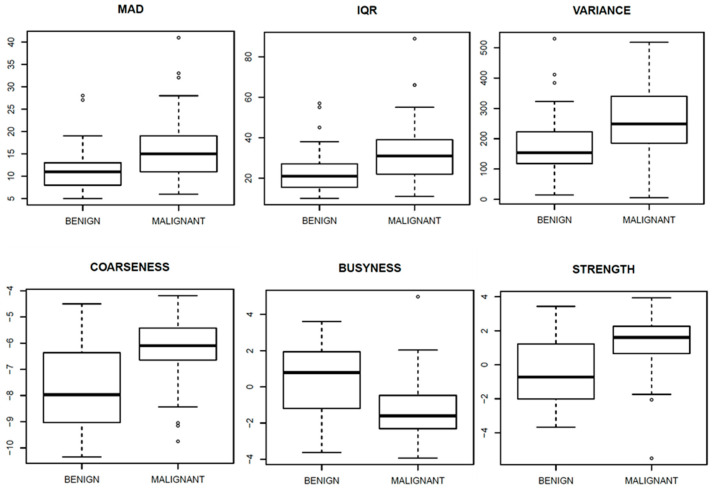
Boxplot of significant textural features for CC view.

**Figure 7:**
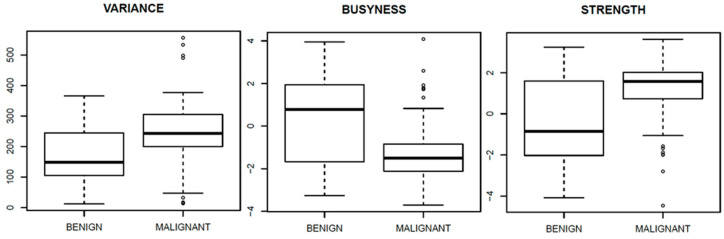
Boxplot of significant textural features for early-MLO view.

**Figure 8:**
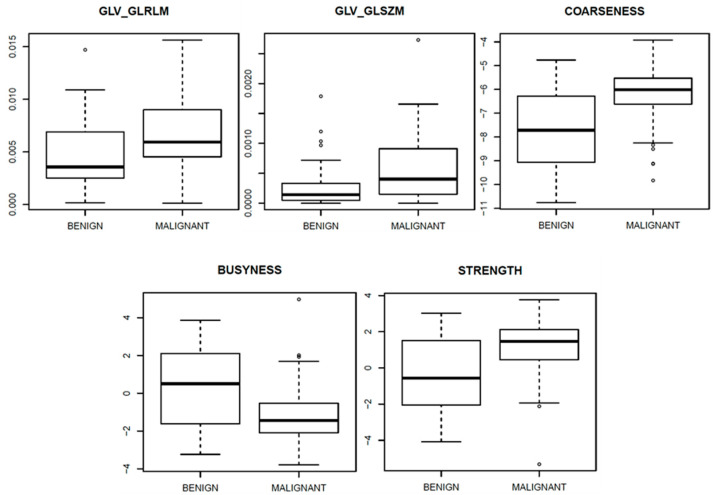
Boxplot of significant textural features for late-MLO view.

Figure 6, Figure 7 and Figure 8 show the boxplots, related to above-mentioned parameters, to separate benign from malignant lesions.

As regard multivariate analysis, only the most useful results for the purposes of this work will be reported. Table 5, Table 6, Table 7 and Table 8 report the performance achieved by best classifiers to discriminate benign from malignant lesions.

The best performance considering the CC view (ACC = 0.75; SENS = 0.75; SPEC = 0.74; PPV = 0.75; NPV = 0.74; AUC = 0.82) was reached with a DT trained with LOOCV and balanced data (with ADASYN function) and a subset of three features (by LASSO and λ_minMSE_). The subset of three robust textural features includes MAD, VARIANCE and LRLGE.

The best performance considering the early-MLO (ACC = 0.77; SENS = 0.80; SPEC = 0.73; PPV = 0.75; NPV = 0.78; AUC = 0.83) was reached with a NNET trained with LOOCV and balanced data (with ADASYN function) and a subset of ten features (by LASSO and λ_minMSE_). The subset of ten robust textural features includes MEAN, MAD, RANGE, IQR, VARIANCE, CORRELATION, RLV, COARSNESS, BUSYNESS, and STRENGTH.

The best performance considering the late-MLO (ACC = 0.73; SENS = 0.74; SPEC = 0.71; PPV = 0.71; NPV = 0.75; AUC = 0.82) was reached with an NNET trained with LOOCV and balanced data (with ADASYN function) and a subset of eleven features (by LASSO). The subset of eleven robust textural features includes MODE, MEDIAN, RANGE, RLN, LRLGE, RLV, LZLGE, GLV_GLSZM, ZSV, COARSNESS, and BUSYNESS.

However, the best result (ACC = 0.87; SENS = 0.86; SPEC = 0.87; PPV = 0.88; NPV = 0.86; AUC = 0.90), were obtained considering 144 textural features extracted from all three mammographic projections (CC, early MLO, and late MLO) at the same time with an SVM trained with LOOCV and with balanced data (with ADASYN function).

**Figure 9:**
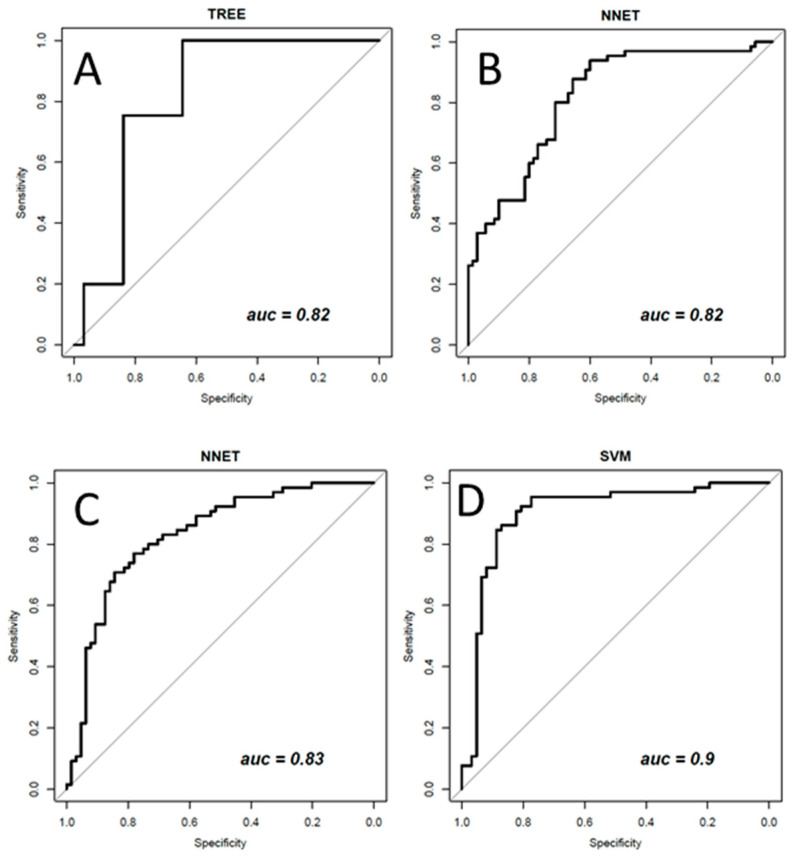
ROC curve of the best classifier in (**A**) a TREE using a subset of three robust textural features extracted by CC view; in (**B**) a NNET using a subset of ten robust textural features extracted by early-MLO view; in (**C**) an NNET using a subset of eleven robust textural features extracted by late-MLO view; in (**D**) a SVM using the all-balanced textural features set extracted from all three mammographic projections (CC, early MLO, and late MLO).

Figure 9 shows the ROC curve of the best classifiers.

## 4. Discussion and Conclusions

Numerous studies have demonstrated increased sensitivity and specificity of CEM compared MX, FFDM, Ultrasound, or Digital Breast Tomosynthesis (DBT). In addition, CEM rivals the sensitivity of more costly and time-consuming examinations, such as MRI and molecular breast imaging [9,11,14,53,54,55,56,57,58,59].

Studies of CEM focused on imaging analysis with an auxiliary system for reporting, and on radiomics features for benignant and malignant differentiation [60,61].

The radiomics analysis of tumor features extracted from CEM images combined with qualitative and quantitative information on morphology and functionality represents an important tool for breast tumor characterization [60,61,62,63]. A summary data was provided in the Table 9 in order to report the main findings using radiomic and artificial intelligence analysis on CEM.

Marino et al. [62] investigated the potential of CEM and radiomics analysis for the noninvasive differentiation of breast cancer invasiveness, hormone receptor status, and tumor grade. Radiomics features were derived from first-order histogram (HIS), co-occurrence matrix (COM), run length matrix (RLM), absolute gradient, autoregressive model, the discrete Haar wavelet transform (WAV), and lesion geometry. They reported that radiomics analysis with CEM has potential for noninvasive differentiation of tumors with different degrees of invasiveness, hormone receptor status, and tumor grade.

La Forgia et al. [63] evaluated radiomics features for predicting histological outcome and two cancer molecular subtypes, namely, Human Epidermal growth factor Receptor 2 (HER2)-positive and triple-negative suggesting an interesting role for radiomics in CEM to predict histological outcomes and particular tumors’ molecular subtype.

In this study, we aimed to evaluate radiomics analysis with texture features extracted by dual-energy CEM images in the classification of malignant and benign breast. We performed the evaluation considering both a univariate analysis and a multivariate analysis using pattern recognition approaches.

The univariate analysis on features extracted from CC view shows statistically positive results for MAD (AUC = 0.72), IQR (AUC = 0.71) and VARIANCE (AUC = 0.72) among first order gray-level statistics, in addition to COARSNESS, BUSYNESS and STRENGTH as regards Neighborhood Gray-Tone Difference Matrix. Considering these results, the univariate analysis showed low accuracy and area under the curve for all considered features in the differentiation of benign and malignant lesions.

As regards multivariate analysis, using the unbalanced dataset showed no significant results; in particular, specificity assumed low values. After a balancing operation (with ADASYN function) and a features selection operation (with LASSO approach) higher values of accuracy, specificity and AUC were measured. A TREE trained with balanced data and a subset of three robust textural predictors (MAD, VARIANCE and LRLGE) achieved the best performance with an accuracy of 0.75 and an AUC of 0.82.

As regards the early MLO view univariate analysis, higher AUC values occurred in correspondence with VARIANCE, BUSYNESS and STRENGTH (AUC = 0.70 in all three cases). With the multivariate analysis, a balancing operation (both with the ADASYN function and the SASYNO function) and a features selection operation allowed to reach good improvements. The best performance (accuracy of 0.77 and AUC of 0.83) was obtained with a NNET trained with balanced data (with ADASYN function) and a subset of robust textural features including MEAN, MAD, RANGE, IQR, VARIANCE, CORRELATION, RLV, COARSNESS, BUSYNESS, and STRENGTH.

With the univariate analysis on features extracted from late MLO view, significant results were measures in correspondence of COARSNESS (AUC = 0.74), BUSYNESS (AUC = 0.71), and STRENGTH (AUC = 0.70), in addition to GLV as regards both the Gray-Level Run-Length Matrix and the Gray-Level Size Zone Matrix (AUC values of 0.70 in either case).

As for the multivariate analysis, the best performance (accuracy of 0.73 and AUC of 0.82) using late MLO features was reached with an NNET trained with balanced data (with ADASYN function) and a subset of robust textural features including MODE, MEDIAN, RANGE, RLN, LRLGE, RLV, LZLGE, GLV_GLSZM, ZSV, COARSNESS and BUSYNESS.

Presently, the determination of the breast malignancies is performed with biopsy. Unfortunately, this examination is invasive and then, there is great interest in alternative, non-invasive and cheaper methods to derive the same information directly from the radiologic images. Another important observation of automated systems for radiomics analysis is that these are not affected by factors such as breast density or background parenchymal enhancement (BPE), which may be limiting breast lesions differentiation. Consequently, these systems also preserve their reliability in more complex breast investigations. Therefore, also that textural analyses is again little used in clinical routine practice; in this context, the radiomics analysis of tumor textural features extracted from CEM images represent an important robust tool for breast tumor characterization.

The small cohort of studied patients represents an initial finding to validate by increasing the sample size of the study in the future. The segmentation of the ROIs slice by slice was manual, and this can be time-consuming, can be operator variable, can be difficult in the segmentation of multicentric lesions or in cases of background parenchymal enhancement. In this study is not reported a comparison of accuracy in the breast lesions detection of CEM respect to FFDM. The future endpoint is to include in the analysis an automatic segmentation of the lesions. The radiomics analysis did not consider tumor histological differences, while the integration of texture metrics combined with histopathology results may provide other important prognostic information for the classification of malignant breast lesions both in early and late phase. This could improve the performance in the classification problem and allow classifying breast lesions according grading and histotype. We did not evaluate the possible impact on texture values considering the different impact of the “void sign” after VAAB vs. post FNAC. This could be evaluated in a future study.

In conclusion, multivariate analyses using pattern recognition approaches optimized by adaptive synthetic sampling and feature selection operations obtained the best results to separate benign and malignant lesions. Overall, textural features extracted from CC view images, showed the best performance. However, the best results (ACC = 0.87; AUC = 0.90) were reached by considering all textural features extracted from dual-energy CEM images, using an SVM trained with balanced data. These findings represented a preliminary experience that should be validate with a larger sample size including patients by different centers.

## Figures and Tables

**Table 1:** Number and corresponding percentage of the total benign or malignant breast lesions.

Benign (39 Lesions)	Number	Percentage Value (%)
Fibrosis	8	20.51
Ductal hyperplasia	10	25.64
Fibroadenoma	13	33.33
Dysplasia	4	10.26
Adenosis	4	10.26
**Malignant (65 Lesions)**	**Number**	**Percentage Value (%)**
Infiltrating lobular carcinoma	8	12.31
Infiltrating ductal carcinoma	36	52.17
Ductal carcinoma in situ	12	18.46
Intraductal papilloma with DCIS component	2	3.08
Tubular carcinoma	3	4.61
Papillary carcinoma	2	3.08
Mucinous Carcinoma	2	3.08

**Table 2:** Extracted textural features.

Feature Name	Acronym
**1st Order Gray-Level Statistics**	
Mean	-
Mode	-
Median	-
Standard Deviation	STD
Median Absolute Deviation	MAD
Range	-
Kurtosis	-
Interquartile Range	IQR
Variance	-
Skewness	-
**Gray-Level Co-Occurrence Matrix (GLCM)**	
Energy	-
Contrast	-
Entropy	-
Homogeneity	-
Correlation	-
Sum Average	-
Dissimilarity	-
Autocorrelation	-
**Gray-Level Size Zone Matrix (GLSZM)**	
Small Zone Emphasis	SZE
Large Zone Emphasis	LZE
Gray-Level Non-uniformity	GLN_ GLSZM
Zone-Size Non-uniformity	ZSN
Zone Percentage (ZP)	ZP
Low Gray-Level Zone Emphasis	LGZE
High Gray-Level Zone Emphasis	HGZE
Small Zone Low Gray-Level Emphasis	SZLGE
Small Zone High Gray-Level Emphasis	SZHGE
Large Zone Low Gray-Level Emphasis	LZLGE
Large Zone High Gray-Level Emphasis	LZHGE
Gray-Level Variance	GLV_GLSZM
Zone-Size Variance	ZSV
**Neighborhood Gray-Tone Difference Matrix (NGTDM)**	
Coarseness	-
Busyness	-
Complexity	-
Strength	-

**Table 3:** Configuration settings for each classifier.

Classifier	Configuration Settings
LDA	Covariance structure: full; optimizer options: hyperparameter options disabled
Decision tree	Fine Tree; maximum number of splits: 100; split criterion: Gini’s diversity index; surrogate decision splits: off; optimizer options: hyperparameter options disabled
Artificial neural network	Hidden = 3; linear output to TRUE; optimizer options: hyperparameter options disabled
K-nearest neighbors	Fine KNN; number of neighbors: 100; distance metric: Euclidean; distance weight: equal; standardize data: true; optimizer options: hyperparameter options disabled
Support vector machine	Linear SVM; kernel function: linear; kernel scale: automatic; box constraint level: 1; multiclass method: one-vs-one; standardize data: true; optimizer options; hyperparameter options disabled

**Table 4:** List of most significant textural features with the corresponding area under curve (AUC) and *p*-value.

Mammography Projection	Textural Parameters	AUC Values	*p*-Value
**CC view**	MAD	0.72	0.000
	IQR	0.71	0.000
	VARIANCE	0.72	0.000
	COARSNESS	0.73	0.000
	BUSYNESS	0.73	0.000
	STRENGTH	0.74	0.000
**early MLO view**	VARIANCE	0.70	0.001
	BUSYNESS	0.70	0.001
	STRENGTH	0.70	0.000
**late MLO view**	GLV_GLRLM	0.70	0.001
	GLV_GLSZM	0.70	0.001
	COARSNESS	0.74	0.000
	BUSYNESS	0.71	0.000
	STRENGTH	0.70	0.001

**Table 5:** Performance of the best classifier using textural features extracted from CC view.

Classifier	Cross-Validation	ACC	SENS	SPEC	PPV	NPV	AUC
Performance for classifiers trained with balanced data (with ADASYN function) and a set of 3 textural features (by LASSO and λ_1SE_).							
TREE	LOOCV	0.75	0.75	0.74	0.75	0.74	**0.82**
SVM	LOOCV	0.76	0.77	0.74	0.76	0.75	0.81
Performance for classifiers trained with balanced data (with ADASYN function) and subset of 34 textural features (AUC ≥ 0.60).							
LDA	LOOCV	0.79	0.72	0.85	0.84	0.75	0.80

**Table 6:** Performance of the best classifier using textural features extracted from early-MLO view.

Classifier	Cross-Validation	ACC	SENS	SPEC	PPV	NPV	AUC
Performance for classifiers trained with balanced data (with SASYNO function) and a set of 23 robust textural features (by LASSO and λ_minMSE_).							
SVM	*10*-fold CV	0.79	0.80	0.77	0.78	0.79	0.80
Performance for classifiers trained with balanced data (with SASYNO function) and a subset of 15 robust textural features (AUC ≥ 0.60).							
KNN	*10*-fold CV	0.78	0.77	0.77	0.77	0.77	0.81
Performance for classifiers trained with balanced data (with ADASYN function) and a set of 10 robust textural features (by LASSO and λ_minMSE_).							
KNN	*10*-fold CV	0.78	0.75	0.80	0.79	0.76	0.81
NNET	LOOCV	0.77	0.8	0.73	0.75	0.78	**0.83**

**Table 7:** Performance of the best classifier using textural features extracted from late-MLO view.

Classifier	Cross-Validation	ACC	SENS	SPEC	PPV	NPV	AUC
Performance for classifiers trained with balanced data (with ADASYN function) and all 48 textural features.							
LDA	*10*-fold CV	0.78	0.71	0.84	0.81	0.76	0.79
Performance for classifiers trained with balanced data (with ADASYN function) and a subset of 11 robust textural features (by LASSO).							
NNET	*10*-fold CV	0.74	0.77	0.70	0.70	0.77	0.78
	LOOCV	0.73	0.74	0.71	0.71	0.75	**0.82**

**Table 8:** Performance of the best classifier using textural features extracted from all three mammographic projection (CC, early MLO and late MLO).

Classifier	Cross-Validation	ACC	SENS	SPEC	PPV	NPV	AUC
Performance for classifiers trained with balanced data (with ADASYN function) and all 144 features.							
SVM	LOOCV	0.87	0.86	0.87	0.88	0.86	**0.90**
Performance for classifiers trained with balanced data (with SASYNO function) and a subset of 18 robust textural features (by LASSO and λ_minMSE_).							
LDA	*10*-fold CV	0.85	0.83	0.86	0.86	0.84	0.89
	LOOCV	0.85	0.85	0.86	0.86	0.85	0.89
Performance for classifiers trained with balanced data (with ADASYN function) and set of 13 robust textural features (by LASSO and λ_1SE_).							
LDA	*10*-fold CV	0.84	0.83	0.85	0.84	0.83	0.89
	LOOCV	0.84	0.85	0.83	0.83	0.84	0.89

**Table 9:** Summary table of main findings using radiomic and artificial intelligence analysis on CEM.

Reference	Extracted Features	Used Classifier	Results
[60]	Statistical measures on original ROIs, gradiented images, Haar decompositions of the same original ROIs, and on gray-level co-occurrence matrices of each sub-ROI obtained by Haar transform.	SVM classifier	The features extracted from each sub-ROI decomposed by two levels of Haar transform were predictive only when they were all used without any selection, reaching the best mean accuracy of about 80%.
[61]	Statistical Features	Random Forest classifier	The present method resulted highly performing in the prediction of benign/malignant ROIs with median values of sensitivity and specificity of 87.5% and 91.7%, respectively. The classification model outperformed the human reader, by increasing the specificity over 8%.
[62]	Radiomic features were derived from first-order HIS, COM, RLM, absolute gradient, autoregressive model, the discrete Haar WAV, and lesion geometry. Fisher, probability of error and average correlation (POE + ACC), and mutual information (MI) coefficients informed feature selection.	Linear discriminant analysis followed by k-nearest neighbour classification.	Radiomics analysis achieved the highest accuracies of 87.4% for differentiating invasive from non-invasive cancers based on COM + HIS/MI, 78.4% for differentiating HR positive from HR negative cancers based on COM + HIS/Fisher, 97.2% for differentiating HER2-positive/HR-negative from HER2-negative/HR-positive cancers based on RLM + WAV/MI, 100% for differentiating triple-negative from triple-positive breast cancers mainly based on COM + WAV + HIS/POE + ACC, and 82.1% for differentiating triple-negative from HR-positive cancers mainly based on WAV + HIS/Fisher. Accuracies for differentiating grade 1 vs. grades 2 and 3 cancers were 90% for invasive cancers (based on COM/MI) and 100% for non-invasive cancers (almost entirely based on COM/MI).
[63]	Fourteen statistical features	Multivariate Linear Discriminant Analysis	The highest performances were obtained for discriminating HER2+/HER2− (90.87%), ER+/ER− (83.79%) and Ki67+/Ki67− (84.80%).
Our Study	48 textural parameters were extracted by manually segmenting regions of interest.	Linear classifier, decision tree, k-nearest neighbours, artificial neural network and support vector machine were utilized.	Multivariate analyses considering 144 textural features extracted from all three mammographic projections (CC, early MLO and late MLO) optimized by adaptive synthetic sampling and feature selection operations obtained the best results (ACC = 0.87; AUC = 0.90) and showed the best performance in the discrimination of benign and malignant lesions.

## Data Availability

All data are reported in the manuscript.

## Appendix A. Definition of Textural Features

**First-Order Gray-Level Statistics**

First order gray-level statistics describe the distribution of gray-values within the volume. Let *X* denote the 3-D image matrix with *N* voxels, *P* the first order histogram, *P*(*i*) the fraction of voxels with intensity level *i* and *N*l the number of discrete intensity levels.

- **Mean**, the mean gray-level of X.
  $$mean=\frac{1}{N}\overset{N}{\underset{i=1}{\sum }}X\left(i\right)$$
- **Mode**, the most frequent element(s) of array X.
- **Median**, the sample median of X, or the 50th percentile of X.
- Standard deviation (STD)
  $$STD={\left(\frac{1}{N-1}\overset{N}{\underset{i=1}{\sum }}{\left(X\left(i\right)-\bar{X}\right)}^{2}\right)}^{1/2}$$
- **Mean Absolute Deviation** (**MAD**), the mean of the absolute deviation of all voxel intensities around the mean intensity value.
  $$MAD=\frac{1}{N}\overset{N}{\underset{i=1}{\sum }}\left|X\left(i\right)-\bar{X}\right|$$
- **Range**, the range of intensity values of X.
  range=max(X)−min(X)
  where max(X) is the maximum intensity value of X and min(X) is the minimum intensity value of X.
- **Interquartile range** (**IQR**), the interquartile range is defined as the 75th minus the 25th percentile of X.
- Kurtosis
  $$kurtosis=\frac{\frac{1}{N}\sum_{i=1}^{N}{\left(X\left(i\right)-\bar{X}\right)}^{4}}{{\left(\sqrt{\frac{1}{N}\sum_{i=1}^{N}{\left(X\left(i\right)-\bar{X}\right)}^{2}}\right)}^{2}}$$
  where $\bar{X}$ is the mean of X.
- **Variance,** Variance is the square of the standard deviation.
  $$variance=\frac{1}{N-1}\overset{N}{\underset{i=1}{\sum }}{\left(X\left(i\right)-\bar{X}\right)}^{2}$$
  where $\bar{X}$ is the mean of X.
- **Skewness**
  $$skewness=\frac{\frac{1}{N}\sum_{i=1}^{N}{\left(X\left(i\right)-\bar{X}\right)}^{3}}{{\left(\sqrt{\frac{1}{N}\sum_{i=1}^{N}{\left(X\left(i\right)-\bar{X}\right)}^{2}}\right)}^{3}}$$
  where $\bar{X}$ is the mean of X.

**Gray-Level Co-Occurrence Matrix (GLCM)**

A normalized GLCM is defined as P(i,j;δ,α), a metric with size $N_{g}\times N_{g}$ describing the second-order joint probability function of an image, where the (i,j)th element represents the number of times the combination of intensity levels i and j occur in two pixels in the image, that are separated by a distance of δ pixels in direction α, and $N_{g}$ is the maximum discrete intensity level in the image. Let:

- P(i,j) be the normalized (i.e., ∑P(i,j)=1) co-occurrence matrix, generalized for any δ and α,
- $p_{x}\left(i\right)=\sum_{j=1}^{N_{g}}P\left(i,j\right)$,
- $p_{y}\left(j\right)=\sum_{i=1}^{N_{g}}P\left(i,j\right)$,
- $\mu_{x}$ be the mean of $p_{x}$, where $\mu_{x}=\sum_{i=1}^{N_{g}}\sum_{j=1}^{N_{g}}iP\left(i,j\right)$,
- $\mu_{y}$ be the mean of $p_{y}$, where $\mu_{y}=\sum_{i=1}^{N_{g}}\sum_{j=1}^{N_{g}}jP\left(i,j\right)$,
- $\sigma_{x}$ be the standard deviation of $p_{x}$, where $\sigma_{x}=\sum_{i=1}^{N_{g}}\sum_{j=1}^{N_{g}}P\left(i,j\right){\left(i-\mu_{x}\right)}^{2}$,
- $\sigma_{y}$ be the standard deviation of $p_{y}$, where $\sigma_{y}=\sum_{i=1}^{N_{g}}\sum_{j=1}^{N_{g}}P\left(i,j\right){\left(j-\mu_{y}\right)}^{2}$.

- **Energy**
  $$energy=\overset{N_{g}}{\underset{i=1}{\sum }}\overset{N_{g}}{\underset{j=1}{\sum }}{\left[P\left(i,j\right)\right]}^{2}$$
- **Contrast**
  $$contrast=\overset{N_{g}}{\underset{i=1}{\sum }}\overset{N_{g}}{\underset{j=1}{\sum }}{\left|i-j\right|}^{2}P\left(i,j\right)$$
- **Entropy**
  $$entropy=-\overset{N_{g}}{\underset{i=1}{\sum }}\overset{N_{g}}{\underset{j=1}{\sum }}P\left(i,j\right)log_{2}\left[P\left(i,j\right)\right]$$
- **Homogeneity**
  $$homogeneity=\overset{N_{g}}{\underset{i=1}{\sum }}\overset{N_{g}}{\underset{j=1}{\sum }}\frac{P\left(i,j\right)}{1+\left|i-j\right|}\;$$
- **Correlation**
  $$correlation=\frac{\sum_{i=1}^{N_{g}}\sum_{j=1}^{N_{g}}ijP\left(i,j\right)-\mu_{x}\mu_{y}}{\sigma_{x}\sigma_{y}}$$
- **Sum Average**
  $$sum\;average=\frac{1}{N_{g}\times N_{g}}\overset{N_{g}}{\underset{i=1}{\sum }}\overset{N_{g}}{\underset{j=1}{\sum }}\left[iP\left(i,j\right)+jP\left(i,j\right)\right]$$
- **Dissimilarity**
  $$dissimilarity=\overset{N_{g}}{\underset{i=1}{\sum }}\overset{N_{g}}{\underset{j=1}{\sum }}\left|i-j\right|P\left(i,j\right)\;$$
- **Autocorrelation**
  $$autocorrelation=\overset{N_{g}}{\underset{i=1}{\sum }}\overset{N_{g}}{\underset{j=1}{\sum }}ijP\left(i,j\right)\;$$

**Gray-Level Run-Length Matrix (GLRLM)**

Run length metrics quantify gray level runs in an image. A gray level run is defined as the length in number of pixels, of consecutive pixels that have the same gray level value. In a gray level run length matrix p(i,j|θ), the (i,j)th element describes the number of times *j* a gray level *i* appears consecutively in the direction specified by *θ*. Let:

- p(i,j) be the (i,j)th entry in the given run-length matrix *p*, generalized for any direction *θ*,
- $N_{g}$ the number of discrete intensity values in the image,
- $N_{r}$ the maximum run length,
- $N_{s}$ the total numbers of runs, where $N_{s}=\sum_{i=1}^{N_{g}}\sum_{j=1}^{N_{r}}p\left(i,j\right)$
- $p_{r}$ the sum distribution of the number of runs with run length j, where $p_{r}\left(j\right)=\sum_{i=1}^{N_{g}}p\left(i,j\right)$,
- $p_{g}$ the sum distribution of the number of runs with run length i, where $p_{g}\left(i\right)=\sum_{j=1}^{N_{r}}p\left(i,j\right)$,
- $N_{p}$ the number of voxels in the image, where $N_{p}=\sum_{j=1}^{N_{r}}jp_{r}$,
- $\mu_{r}$ the mean run length, where $\mu_{r}=\sum_{i=1}^{N_{g}}\sum_{j=1}^{N_{r}}jp_{n}\left(i,j\right)$,
- $\mu_{g}$ the mean gray level, where $\mu_{g}=\sum_{i=1}^{N_{g}}\sum_{j=1}^{N_{r}}ip_{n}\left(i,j\right)$.

- **Short Run Emphasis (SRE)**
  $$SRE=\overset{N_{r}}{\underset{j=1}{\sum }}\frac{p_{r}}{j^{2}}$$
- **Long Run Emphasis (LRE)**
  $$LRE=\overset{N_{r}}{\underset{j=1}{\sum }}j^{2}p_{r}$$
- **Gray-Level Nonuniformity (GLN)**
  $$GLN=\overset{N_{g}}{\underset{i=1}{\sum }}p_{g}{}^{2}$$
- **Run-Length Nonuniformity (RLN)**
  $$RLN=\overset{N_{r}}{\underset{j=1}{\sum }}p_{r}{}^{2}$$
- **Run Percentage (RP)**
  $$RP=\frac{N_{s}}{N_{p}}$$
- **Low Gray-Level Run Emphasis (LGRE)**
  $$LGRE=\overset{N_{g}}{\underset{i=1}{\sum }}\frac{p_{g}}{i^{2}}$$
- **High Gray-Level Run Emphasis (HGRE)**
  $$HGRE=\overset{N_{g}}{\underset{i=1}{\sum }}i^{2}p_{g}$$
- **Short Run Low Gray-Level Emphasis (SRLGE)**
  $$SRLGE=\overset{N_{g}}{\underset{i=1}{\sum }}\overset{N_{r}}{\underset{j=1}{\sum }}\frac{p\left(i,j\right)}{i^{2}j^{2}}$$
- **Short Run High Gray-Level Emphasis (SRHGE)**
  $$SRHGE=\overset{N_{g}}{\underset{i=1}{\sum }}\overset{N_{r}}{\underset{j=1}{\sum }}\frac{p\left(i,j\right)i^{2}}{j^{2}}$$
- **Long Run Low Gray-Level Emphasis (LRLGE)**
  $$LRLGE=\overset{N_{g}}{\underset{i=1}{\sum }}\overset{N_{r}}{\underset{j=1}{\sum }}\frac{p\left(i,j\right)j^{2}}{i^{2}}$$
- **Long Run High Gray-Level Emphasis (LRHGE)**
  $$LRHGE=\overset{N_{g}}{\underset{i=1}{\sum }}\overset{N_{r}}{\underset{j=1}{\sum }}p\left(i,j\right)i^{2}j^{2}$$
- **Gray-Level Variance (GLV)**
  $$GLV=\frac{1}{N_{g}\times N_{r}}\;\overset{N_{g}}{\underset{i=1}{\sum }}\overset{N_{r}}{\underset{j=1}{\sum }}{\left(ip\left(i,j\right)-\mu_{g}\right)}^{2}$$
- **Run-Length Variance (RLV)**
  $$RLV=\frac{1}{N_{g}\times N_{r}}\;\overset{N_{g}}{\underset{i=1}{\sum }}\overset{N_{r}}{\underset{j=1}{\sum }}{\left(jp\left(i,j\right)-\mu_{r}\right)}^{2}$$

**Gray-Level Size Zone Matrix (GLSZM)**

A gray level size-zone matrix describes the amount of homogeneous connected areas within the volume, of a certain size and intensity. The (i,j)th entry of the GLSZM p(i,j) is the number of connected areas of gray-level (i.e., intensity value) *i* and size *j*. GLSZM features therefore describe homogeneous areas within the tumor volume, describing tumor heterogeneity at a regional scale [5]. Let:

- p(i,j) be the (i,j)th entry in the given GLSZM p,
- $N_{g}$ the number of discrete intensity values in the image,
- $N_{z}$ the size of the largest, homogeneous region in the volume of interest,
- $N_{s}$ the total number of homogeneous regions (zones), where $N_{s}=\sum_{i=1}^{N_{g}}\sum_{j=1}^{N_{z}}p\left(i,j\right)$
- $p_{z}$ the sum distribution of the number of zones with size j, where $p_{z}\left(j\right)=\sum_{i=1}^{N_{g}}p\left(i,j\right)$,
- $p_{g}$ the sum distribution of the number of zones with gray level i, where $p_{g}\left(i\right)=\sum_{j=1}^{N_{z}}p\left(i,j\right)$,
- $N_{p}$ the number of voxels in the image, where $N_{p}=\sum_{j=1}^{N_{z}}jp_{r}$,
- $\mu_{r}$ the mean zone size, where $\mu_{r}=\sum_{i=1}^{N_{g}}\sum_{j=1}^{N_{z}}jp(i,j|\theta )$,
- $\mu_{g}$ the mean gray level, where $\mu_{g}=\sum_{i=1}^{N_{g}}\sum_{j=1}^{N_{z}}ip(i,j|\theta )$.

- **Small Zone Emphasis (SZE)**
  $$SZE=\overset{N_{z}}{\underset{j=1}{\sum }}\frac{p_{z}}{j^{2}}$$
- **Large Zone Emphasis (LZE)**
  $$LZE=\overset{N_{z}}{\underset{j=1}{\sum }}j^{2}p_{z}$$
- **Gray-Level Non-uniformity (GLN)**
  $$GLN=\overset{N_{g}}{\underset{i=1}{\sum }}p_{g}{}^{2}$$
- **Zone-Size Non-uniformity (ZSN)**
  $$ZSN=\overset{N_{g}}{\underset{i=1}{\sum }}p_{z}{}^{2}$$
- **Zone Percentage (ZP)**
  $$ZP=\frac{N_{s}}{N_{p}}$$
- **Low Gray-Level Zone Emphasis (LGZE)**
  $$LGZE=\overset{N_{g}}{\underset{i=1}{\sum }}\frac{p_{g}}{i^{2}}$$
- **High Gray-Level Zone Emphasis (HGZE)**
  $$HGZE=\overset{N_{g}}{\underset{i=1}{\sum }}i^{2}p_{g}$$
- **Small Zone Low Gray-Level Emphasis (SZLGE)**
  $$SZLGE=\overset{N_{g}}{\underset{i=1}{\sum }}\overset{N_{z}}{\underset{j=1}{\sum }}\frac{p\left(i,j\right)}{i^{2}j^{2}}$$
- **Small Zone High Gray-Level Emphasis (SZHGE)**
  $$SZHGE=\overset{N_{g}}{\underset{i=1}{\sum }}\overset{N_{z}}{\underset{j=1}{\sum }}\frac{p\left(i,j\right)i^{2}}{j^{2}}$$
- **Large Zone Low Gray-Level Emphasis (LZLGE)**
  $$LZLGE=\overset{N_{g}}{\underset{i=1}{\sum }}\overset{N_{z}}{\underset{j=1}{\sum }}\frac{p\left(i,j\right)j^{2}}{i^{2}}$$
- **Large Zone High Gray-Level Emphasis (LZHGE)**
  $$LZHGE=\overset{N_{g}}{\underset{i=1}{\sum }}\overset{N_{z}}{\underset{j=1}{\sum }}p\left(i,j\right)j^{2}i^{2}$$
- **Gray-Level Variance (GLV)**
  $$GLV=\frac{1}{N_{g}\times N_{z}}\;\overset{N_{g}}{\underset{i=1}{\sum }}\overset{N_{z}}{\underset{j=1}{\sum }}{\left(ip\left(i,j\right)-\mu_{g}\right)}^{2}$$
- **Zone-Size Variance (ZSV)**
  $$ZSV=\frac{1}{N_{g}\times N_{z}}\;\overset{N_{g}}{\underset{i=1}{\sum }}\overset{N_{z}}{\underset{j=1}{\sum }}{\left(jp\left(i,j\right)-\mu_{z}\right)}^{2}$$

**Neighborhood Gray Tone Difference Matrix (NGTDM)**

The *i*th entry of the NGTDM s(i|d) is the sum of gray level differences of voxels with intensity *i* and the average intensity $A_{i}$ of their neighboring voxels within a distance *d*. Let:

- $n_{i}$ be the number of voxels with gray level i,
- $N=\sum n_{i}$, the total number of voxels,
- $s\left(i\right)=\{\begin{array}{c} \sum_{n_{i}}\left|i-A_{i}\right|\;for\;n_{i}>0\\ 0\;otherwise \end{array}$, generalized for any distance d,
- $N_{g}$ be the maximum discrete intensity level in the image,
- $p\left(i\right)=\frac{n_{i}}{N}$, the probability of gray level i,
- $N_{p}$, the total number of gray levels present in the image.

- **Coarseness**
  $$coarseness={\left[\varepsilon +\overset{N_{g}}{\underset{n=1}{\sum }}p\left(i\right)s\left(i\right)\right]}^{-1}$$
  where ε is a small number to prevent coarseness becoming infinite.
- **Contrast**
  $$contrast=\left(\frac{1}{N_{p}\left(1-N_{p}\right)}\overset{N_{g}}{\underset{i=1}{\sum }}\overset{N_{g}}{\underset{j=1}{\sum }}p\left(i\right)p\left(j\right){\left(i-j\right)}^{2}\right)\left(\frac{1}{N}\overset{N_{g}}{\underset{i=1}{\sum }}s\left(i\right)\right)$$
- **Busyness**
  $$busyness=\frac{\sum_{i=1}^{N_{g}}p\left(i\right)s\left(i\right)}{\sum_{i=1}^{N_{g}}\sum_{j=1}^{N_{g}}\left|ip\left(i\right)-jp\left(j\right)\right|},\;\;\;p\left(i\right)\neq 0,\;p\left(j\right)\neq 0\;$$
- **Complexity**
  $$complexity=\overset{N_{g}}{\underset{i=1}{\sum }}\overset{N_{g}}{\underset{j=1}{\sum }}\left|i-j\right|\frac{p\left(i\right)s\left(i\right)+p\left(j\right)s\left(j\right)}{N\left(p\left(i\right)+p\left(j\right)\right)},\;\;\;p\left(i\right)\neq 0,\;p\left(j\right)\neq 0$$
- **Strength**
  $$strength=\frac{\sum_{i=1}^{N_{g}}\sum_{j=1}^{N_{g}}\left[p\left(i\right)+p\left(j\right)\right]{\left(i-j\right)}^{2}}{\varepsilon +\sum_{n=1}^{N_{g}}s\left(i\right)},\;\;\;p\left(i\right)\neq 0,\;p\left(j\right)\neq 0\;$$
  where ε is a small number to prevent strength becoming infinite.
